# Effects of Water Regime, Genotype, and Formative Stages on the Agro-Physiological Response of Sugarcane (*Saccharum* *officinarum* L.) to Drought

**DOI:** 10.3390/plants9050661

**Published:** 2020-05-23

**Authors:** Jose Arnel O. Reyes, Arvin S. Carpentero, Primitivo Jose A. Santos, Evelyn F. Delfin

**Affiliations:** Institute of Plant Breeding, College of Agriculture and Food Science, University of the Philippines at Los Baños College, Laguna 4031, Philippines; joreyes7@up.edu.ph (J.A.O.R.); arvincarpenter@gmail.com (A.S.C.); pasantos3@up.edu.ph (P.J.A.S.)

**Keywords:** sugarcane, drought, antioxidant activity, electrolyte leakage, formative stages

## Abstract

Drought during the formative stages of a plant’s growth triggers a sequence of responses to maintain optimal growing conditions, but often at the expense of crop productivity. Two field experiments were conducted to determine the effect of drought on 10 high-yielding sugarcane genotypes at two formative stages (the tillering stage (TS) and stalk elongation (SS)), within 30 days after treatment imposition. The experiments followed a split-plot in a randomized complete block design with three replicates per genotype. Agro-physiological responses to drought were observed to compare the differences in the response of sugarcane during the two formative stages. Drought significantly reduced total chlorophyll content (Chl) and stomatal conductance (Gs) for both formative stages, while significantly increasing total scavenging activity (AOA) and electrolyte leakage (EC). A higher level of Chl was observed in the stalk elongation stage compared to the tillering stage; however, lower AOA coupled with higher EC in the stalk elongation stage suggests higher drought susceptibility. Pearson's correlation analysis revealed a stronger correlation between plant height, internode length, Chl, AOA, EC, and Gs at the tillering stage relative to the stalk elongation stage. Moreover, results from the multivariate analysis indicate the different contribution values of each parameter, supplementing the hypothesized difference in response between the two formative stages. Multivariate analysis clustered the 10 genotypes into groups based on the traits evaluated, suggesting the ability of these traits to detect differences in a sample population. The observed relationship among traits during the two formative stages of sugarcane will be significant in screening and identifying drought-susceptible and drought-tolerant genotypes for variety development studies.

## 1. Introduction

Sugarcane (*Saccharum officinarum* L.) is an important crop cultivated globally, mainly for sugar and biofuel. In the Philippines, it contributes approximately 70 billion Philippine pesos (PHP) annually and is currently one of the top agricultural crops in the country, in terms of area harvested (437,070 hectares devoted) and yield (29.29.7 million metric tons), which is equivalent to a national yield average of 67 tons/ha [1]. Variable weather conditions, such as extreme dry spells and excessive rains, were identified as one of the chief causes of low sugarcane production in the Philippines and elsewhere [2]. 

Drought is one of the major abiotic stresses experienced by sugarcane grown under rainfed conditions, causing a negative impact on crop development and productivity. Studies have found that with limited moisture, cane yield and total dry matter can be reduced by 17–52% and 20–56%, respectively [3]. Yield components, such as the number of millable canes, stalk diameter, and cane length, were also adversely affected [4,5,6]. Leaf traits, such as leaf area, leaf number, and leaf area index were also reduced [4,7]. During drought, photosynthesis is highly affected, as is stomatal conductance [7,8,9]. In one study, the monitoring of photosynthetic activities under water deficit showed reduced daily leaf carbon balance due to drought [8]. Recent reports have also shown the ability of photosynthetic machinery to recover after the removal of stress [9].

Since sugar cane is a crop with high water requirements, planting less water-requiring or drought-tolerant varieties is recommended as one of the strategies to meet the challenge of water depletion [10]. The sugarcane varieties cultivated in the Philippines were developed for high cane yield, and disease and pest resistance. Although these varieties have been evaluated under a wide range of environmental conditions, evaluation focused on performance under limiting and excessive moisture conditions has not yet been conducted. The opportunity for varietal improvement for drought tolerance exists as shown by significant differences in varietal response to drought under greenhouse and field conditions, covering both agronomic and physiological parameters [6,11,12,13,14,15,16]. Understanding the different mechanisms that affect the drought response of a crop will be useful in selecting and identifying genotypes with potential drought tolerance.

The sugarcane life cycle consists of distinct growth stages that require a steady water supply to maintain normal growth and development [17]. Earlier studies have shown the impact of limited moisture on agronomic and physiological traits at formative stages [11,18,19,20,21]. These studies have also reported on the differential sensitivity of sugarcane to moisture stress at different growth stages; however, there have been no studies yet on the monitoring of sugarcane response to drought imposed at two formative stages (tillering and stalk elongation) under field conditions. 

Thus, this study compared the differences in the responses of 10 sugarcane varieties to drought at two formative stages (tillering and stalk elongation). The physiological parameters used to evaluate the water treatment (E) (drought stress vs. well-watered) responses of each sugarcane genotype (G) at each formative stage (T) were total chlorophyll content (Chl), total scavenging/antioxidant activity (AOA), electrolyte leakage (EC), and stomatal conductance (Gs). Moreover, the agronomic parameters that were used to evaluate yield were plant height (PH), stalk diameter (SD), internode length (IL), and tiller count (TIL). 

## 2. Results

### 2.1. Analysis of Variance and Mean Comparison

Results showed the significant effects of genotype (G), water treatment (E), and formative stage (T) on the total chlorophyll content (Chl), scavenging activity (AOA), electrolyte leakage (EC), stomatal conductance (Gs), plant height (PH), internode length (IL), stalk diameter (SD), and number of tillers (TIL) (Table 1). G × E interaction was observed only in Gs, EC, AOA, and IL. G × T interaction was seen in all of the physiological traits, as well as IL and TIL. E × T interaction was observed in all the parameters, except for SD and IL. G × E × T interaction was present in all the physiological parameters, and IL. The significant effect of drought stress treatment was observed in all parameters, and the variable response to stress by the different genotypes was demonstrated by the presence of interaction effects as revealed by the Analysis of Variance (ANOVA). 

### 2.2. Total Chlorophyll Content

**Figure 1:**
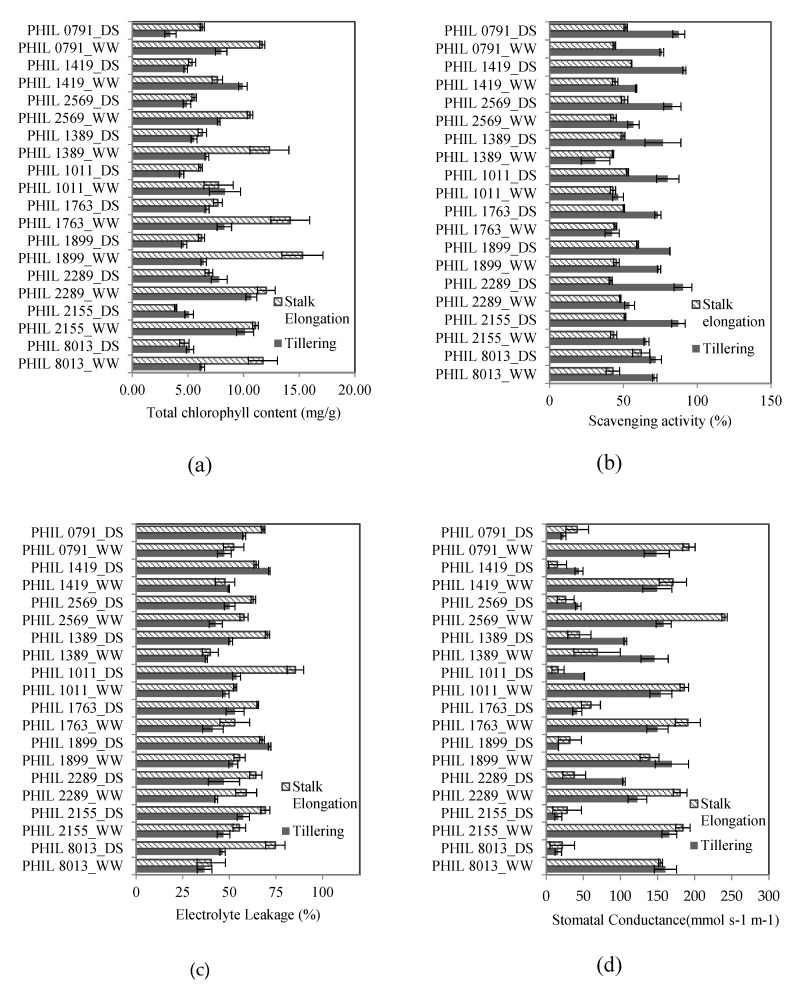
(**a**) Total chlorophyll content; (**b**) scavenging activity; (**c**) electrolyte leakage; and (**d**) stomatal conductance of the 10 sugarcane genotypes subjected to drought during stalk elongation and tillering stages; DS = drought stress; WW = well-watered.

Regarding Chl, significant differences can be observed among sugarcane genotypes. The highest average chlorophyll values can be seen in PHIL 2005-1763 and PHIL 2006-2289 (9.22–9.35 mg/g) while the lowest value was obtained in PHIL 2004-1011 (6.66 mg/g) (Table 1). The average chlorophyll content under the drought stress treatment was significantly lower than that under the well-watered treatment. At the tillering stage, the highest reduction values were seen in PHIL 2000-0791 (57.07%) and 2004-1011 (46.82%), while more stable values were present in PHIL 2003-1389 (17.16%) and PHIL 2005-1753 (18.74%). At the stalk elongation stage, the highest reductions in chlorophyll content were observed in genotypes PHIL 2155 (64.72%) and PHIL 8013 (60.17%), while the lowest reductions were observed in PHIL 2004-1011 (20.96%) and PHIL 2000-1419 (29.58%) (Figure 1a). 

The significant interaction among the three factors evaluated showed that the Chl of each genotype varies in each growing condition and growth stage, and some varieties are quite stable in terms of their Chl values (Table A1). This was evident in the case of PHIL 2005-1763, which had the lowest value among the 10 genotypes in the well-watered and drought stress treatments at the tillering stage and the highest Chl values during drought imposed at the stalk elongation stage. However, other genotypes, such as PHIL 2006-2289, had a relatively stable performance in terms of Chl relative to the other genotypes evaluated (Figure 1a). 

### 2.3. Scavenging/Antioxidant Activity

An effective plant antioxidant system is important in neutralizing the oxidative stress caused by reactive oxygen species (ROS) during unfavorable conditions, resulting in tolerance to multiple abiotic stresses [22]. The 2,2-diphenyl-1-picrylhydrazyl (DPPH) scavenging assay showed significant variation among genotypes in terms of average scavenging (AOA), indicating possible variation in terms of drought tolerance capability. As shown in Table 1, genotypes PHIL 8013 (62.37%), PHIL 2000-1419 (62.61%), PHIL 2000-0791 (64.25%), and PHIL2006-1899 (64.51%) had the highest scavenging values, while genotypes PHIL 2003-1389 (51%) and PHIL 2005-1763 (52.68%) had the lowest. 

There were significant differences in the AOA values between the water treatments and the formative stages, suggesting a difference in the drought sensitivity of each genotype during different formative stages (Table 1). In the drought condition, the highest increase in AOA value was observed in PHIL 2004-1011 at the tillering stage (147.07% increase) followed by PHIL 2004-1011, PHIL 2005-1763, PHIL 2006-2289, and PHIL 2000-1419, which have increases in AOA values above 55.0% (55.86–73.13%) (Figure 1b). At the stalk elongation stage, the highest scavenging activity was observed in PHIL 8013 (44.61%). Interaction between G × E × T was also observed (Figure 1b) (Table A2). In the well-watered condition, PHIL 2005-1763 and PHIL 2003-1389 had higher AOA values at the stalk elongation stage; however, in the drought condition, AOA values of the same genotypes were higher during the tillering stage. This clearly exhibits a differential response of the genotypes during drought at different formative stages. 

### 2.4. Electrolyte Leakage

Electrolyte leakage can be used as an indicator of membrane stability during stress [23]. As expected, significant increases in EC values were observed in plants grown under drought stress treatment (61.78%) compared to the control (47.27%) (Table 1). Variations among sugarcane genotypes, which demonstrate different levels of drought sensitivity, were also present in the experiment, with the highest average values (between 57.60–60.75%) observed in PHIL 2004-1011, PHIL 2000-2569, and PHIL 8013, and the lowest average values (between 48.83–49.08%) observed in PHIL 2000-1419 and PHIL 2006-2289. The percentage increase in EC values was observed to be higher at the stalk elongation stage than in the tillering stage in general (Table 1). However, most of the increase can be attributed to the difference in the EC values (ranging from 60.87% to 85.52%) of PHIL 2000-1419, PHIL 2006-2289, and PHIL2000-2569 at the stalk elongation stage. The increase in leakage values during the tillering stage was highest in PHIL 8013 (43.78%), and the EC values of the rest of the genotypes ranged only from 9.75 to 37.48%. With significant G × E × T interaction, significant genotype differences were observed for both well-watered and drought stress treatment at the stalk elongation stage, whereas significant genotype differences were observed only in the drought stress treatment at the tillering stage (Figure 1c). Genotype ranking also varied for each growth stage and water treatment (Table A3). This observation was most evident in PHIL 2004-1011, which had the highest electrolyte leakage in drought stress treatment at the tillering stage and among genotypes with low electrolyte leakage at the stalk elongation stage.

### 2.5. Stomatal Conductance

The average stomatal conductance (Gs) values for each genotype, as well as between formative stages, were not significantly different from each other (Table 1). This can be attributed to the presence of interaction between the dependent variables (G × E × T). High stomatal resistance has been reported to be advantageous in terms of water economy in a cropping system; however, a steady decline in stomatal conductance could affect growth and yield [18]. A decrease in stomatal conductance is an immediate response of crops to water deficit; therefore, stress recognition and signaling play an important role in stomata regulation. The interaction effects between genotype, water treatment, and formative stage provide evidence of the differences in the effect of drought at each stage for each sugarcane genotype (Table A4). For instance, PHIL 2000-1419 had the highest stomatal conductance at the tillering stage in the drought condition, while PHIL 2003-1389 had the highest at the stalk elongation stage. In terms of genotype performance across water treatments and growth stages, PHIL 2005-1763 showed highly reduced stomatal conductance at the tillering stage, while PHIL 8013 had relatively low conductance at the stalk elongation stage. On the other hand, PHIL 2006-2289 showed low stomatal conductance at both growth stages (Figure 1d). This demonstrates how the same genotypes adjust differently to drought at different formative stages of growth.

### 2.6. Agronomic Traits

In terms of agronomic traits, Table 1 shows that the average plant height of sugarcane genotypes was significantly different from each other. PHIL 2004-1011, PHIL 2155, and PHIL 2005-1763 had the highest values ranging from 115.52 cm to 118.16 cm while the lowest values were observed in PHIL 2000-0791 at 89.45 cm. The internode length of each genotype was also significantly different. The average values of PHIL 2000-2569 (8.46 cm) and PHIL 2000-0791 (9.65 cm) were significantly lower than PHIL 2006-2289 (11.47), PHIL 2004-1011 (11.53 cm), and PHIL 2003-1389 (12.48 cm). Plant heights and internode lengths were significantly different between both water treatments and formative stages, with plants under the well-watered treatment having significantly higher plant height (35.35% difference) and internode length (18.42% difference) compared to those under the drought stress treatment. At the stalk elongation stage, plant height and internode lengths were 40.30% and 31.42% higher than at the tillering stage, respectively. 

The stalk diameter of the genotypes were also significantly different, with the highest average value in PHIL 2155 (33.81 mm) and the lowest value in PHIL 2004-1011 (26.82 mm). Stalk diameter significantly different between water treatments, but not between formative stages, with the well-watered treatments having significantly higher stalk diameter (by 12.96%) than the drought stress treatments. In terms of tiller count, the average values among genotypes showed no significant difference. However, a significant reduction was observed in tiller count in the drought stress treatment (5.24) compared to the control (5.92).

### 2.7. Correlation Analysis of Physiological and Agronomic Traits

Pearson’s correlation analysis was done in order to evaluate the relationships between each physiological trait at the tillering and stalk elongation stages (Table 2 and Table 3). In terms of plant height, a significant correlation was observed for all the physiological parameters for both formative stages—negative correlation for scavenging activity, electrolyte leakage, and stomatal conductance, and positive correlation for total chlorophyll content. Although a similar trend was observed across the physiological parameters, *r*^2^ values suggest a difference in the manner by which drought affects the parameters during the two growth stages (i.e., PH × Chl: *r*^2^ = 0.61, tillering; *r*^2^ = 0.38, stalk elongation; plant height × AOA : *r*^2^ = −0.49, tillering; *r*^2^ = −0.30, stalk elongation; plant height × EC: *r*^2^ =−0.39, tillering; *r*^2^ =−0.43, stalk elongation; plant height × Gs: *r*^2^ = −0.67, tillering; *r*^2^ = −0.51, stalk elongation) (Table 2).

For stalk diameter, both formative stages showed a low correlation between physiological and agronomic parameters. At the tillering stage, a significant positive correlation was observed with Chl (*r*^2^ = 0.38), while a significant negative correlation was present with Gs (*r*^2^ = −0.28). At the stalk elongation stage, a significant negative correlation with AOA (*r*^2^ = −0.38) and Gs (*r*^2^ = −0.26) was observed; however, both relationships were weak (Table 3). 

No significant correlation was observed between internode length and the physiological parameters at the stalk elongation stage. In contrast, in the tillering stage, a significant correlation was present for all the physiological traits. Significant positive correlation was present with chlorophyll content (*r*^2^ = 0.594), while significant negative correlation was present in AOA (*r*^2^ =−0.45), EC (*r*^2^ = −0.41), and Gs (*r*^2^ = −0.58). The significant correlation between tiller count and all physiological parameters was only present at the stalk elongation stage. The *r*^2^ values for each parameter were as follows: Chl (0.52), AOA (−0.33), EC (−0.37), and Gs (−0.45). These results show that drought affects both physiological and agronomic traits differently during both formative stages, which also suggest possible differences in the sensitivity or mechanism of tolerance during each of the growth stages.

### 2.8. Principal Component Analysis and Clustering

The results of principal component analysis (PCA) of genotypes subjected to drought at the tillering and stalk elongation stages are shown in Table 4 and Table 5. In this study, PCA was used to classify each sugarcane genotype into clusters and identify the traits greatly affecting each principal component that were, in turn, used to define the dimension for plotting the data. Therefore, PCs with the highest combined variability contributions were evaluated for both experiments. 

**Figure 2:**
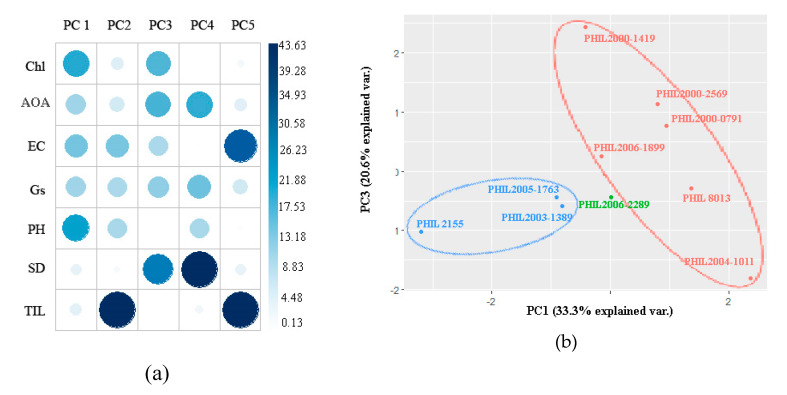
(**a**) Principal component analysis (PCA) showing the different loadings or contributions of each variable per principal component; and (**b**) PCA-based biplot showing the clustering of the 10 sugarcane genotypes subjected to drought at tillering stage using principal component (PC)1 and PC3.

The PCA at the tillering stage revealed that the first four PCs were responsible for 91.6% of the total variation (Table 4). Two parameters (AOA and EC) were positively correlated with PC1, while Chl, Gs, plant height, stalk diameter, internode length, and tiller count were negatively correlated with PC1, which accounts for 33.3% of the variation. For PC3, which contributes 20.6% of the total variance, a positive correlation was present in AOA, Gs, stalk diameter, and tiller count, while a negative correlation was observed in Chl, AOA, and plant height. Despite PC2’s relatively higher variation contribution (24.79), PC3 was selected since it was more balanced and well accounted for. Figure 2a shows the different contributions of each variable for both PC1 and PC3. Chl (22.62), plant height (23.94), and EC (17.79) contribute highly to PC1. For PC3, Chl (20.35), AOA (20.75), Gs (15.31), and stalk diameter (30.82) were the major contributors. 

Biplots based on PC1 and PC3 are shown in Figure 2b. During drought stress at the tillering stage, PHIL 8013, PHIL 2000-2569, PHIL 2000-0791, PHIL 2004-1011, PHIL 2000-1419, and PHIL 2006-1899 were classified by high AOA (82.57%), high EC (59.22%), and high internode length (8.02 mm). PHIL 2155, PHIL 2003-1389, and PHIL 2005-1793 were classified by low Chl (4.57 mg/g), high AOA (82.57%), and high EC (59.22%).

**Figure 3:**
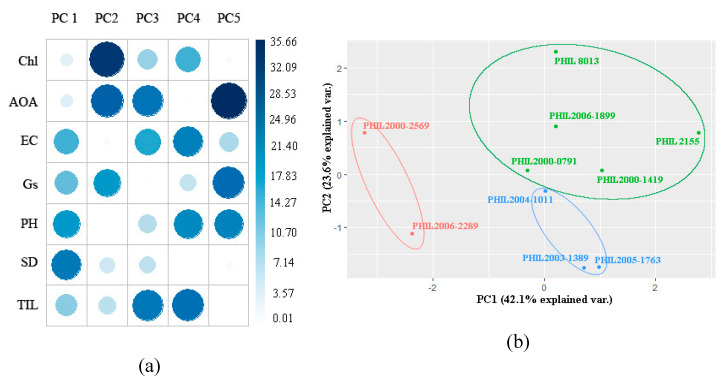
(**a**) Principal component analysis showing the different loadings or contributions of each variable per principal component; (**b**) and PCA-based biplot showing the clustering of the 10 sugarcane genotypes subjected to drought at stalk elongation stage using PC1 and PC2.

The PCA at stalk elongation revealed that the first four PCs accounted for at least 91.10% of the total variation in the data (Table 5). AOA, Gs, plant height, and stalk diameter were positively correlated with PC1, whereas Chl, EC, and tiller count were negatively correlated with PC1. PC1 is responsible for 42.1% of the overall variation of the data set. For PC2, AOA, EC, and plant height were positively correlated, while Chl, Gs, stalk diameter, and tiller count were all negatively correlated. PC2 contributes 23.6% to the overall variation of the data set. Figure 3a shows the relative loadings or contributions of each variable in PC1 and PC2. The majority (78.6%) of the contributions to PC1 were from EC (17.26), Gs (15.33), plant height (20.51), and stalk diameter (25.51; while 83.58% of the total contributions to PC2 came from Chl (34.05), AOA (29.06), and Gs (20.47).

Bi-plots based on PC1 and PC2 (Figure 3b) visualize the relationship of each variety and its apparent grouping using all of the parameters in the study. During drought stress, PHIL 2000-2569 and PHIL 2006-2289 were grouped based on their low AOA (46.09%), low Gs (18.66 mmol m^−2^ s^−1^), high EC (80.05), and stunted plant height (78.85 cm). PHIL 8013, PHIL 2155, PHIL 2000-0791, PHIL 2000-1419, and PHIL 2006-1899 were classified as having the highest AOA (55.93%) and lowest tiller count. The group also has a low EC (67.03) compared to the previous group. Lastly, PHIL 2004-1011, PHIL 2003-1389, and PHIL 2005-1793 were grouped based on their high Chl (6.70 mg/g), high AOA (50.9%), low EC (65.65), high Gs (43.22 mmol m^−2^ s^−1^), and highest plant height value (128.86 cm).

## 3. Discussion

Drought has always been an important factor affecting plant growth. It triggers different biochemical and physiological responses that could alter several morpho-agronomic characteristics related to yield. In the current study, it is hypothesized that the different formative stages of sugarcane have varying sensitivity to drought could be governed by several critical biochemical and physiological traits. Several studies have already compared the response of different crops at different developmental stages of growth, especially between vegetative and reproductive stages. Studies in rice [24], sorghum [25], and wheat [26] suggest that drought at different developmental stages of growth influence both the agronomic and physiological responses of the crop. Critical growth stages (i.e., tillering and stalk elongation) of sugarcane have been identified, both of which are part of the vegetative phase [17]. However, studies comparing the effect of drought at these growth stages on different genotypes have not yet been addressed. Therefore, this study discusses the differences in the agro-physiological response of sugarcane subjected to drought at a particular formative stage, to assist in the identification of tolerant and susceptible sugarcane genotypes. 

Different parameters can be used to evaluate the effects of drought [27,28,29]. Chlorophyll content has been widely used as a parameter for stress evaluation [30]. Several studies have reported the significant effect of drought on the total chlorophyll content in sugarcane. In this study, the average chlorophyll content in the drought stress treatment was significantly lower than the well-watered treatment. Results were similar to other reported studies [27,30], although the present study utilized pigment extraction analysis instead of the SPAD index estimate in order to evaluate chlorophyll content directly. Chlorophyll reduction due to chloroplast pigment and membrane degradation was associated with the production of free radicals during water deficit conditions [29]. Higher chlorophyll content during drought was identified as a good indicator of tolerance because it connotes less chloroplast membrane damage [29]. As a major component of the chloroplast, chlorophyll content has been reported and argued to be correlated with yield maintenance during stressed conditions [31] and, therefore, it may play a vital role in the photosynthetic capability of a crop under stress. 

Higher average chlorophyll content values were observed in plants in the tillering stage (6.76 mg/g) and the stalk elongation stage (8.66 mg/g). Chlorophyll content of most plant species was observed to be significantly lower during its early vegetative stages and increases as it reaches maturity [32]. Moreover, the intense growth stage of sugarcane, which coincides with the tillering stage in this study, was reported to be more susceptible to drought stress [33].

In order to alleviate membrane damage due to free radicals or reactive oxygen species (ROS), an efficient scavenging system to reduce the concentration of these harmful molecules is needed, which also serves as a signaling system to ensure induction of gene expression of different antioxidant enzymes related to drought tolerance [22,34]. This is evident in the higher antioxidant levels of the drought-stressed plants compared to the well-watered plants in this study. The ROS scavenging activity of different sugarcane genotypes was highly dependent on the inherent adaptive mechanism of each variety [35]. In this study, the average scavenging percentage values of each genotype were observed to be significantly different from each other, suggesting possible variation in terms of drought tolerance capability. Sugarcane species with higher levels of scavenging/antioxidant activity through different enzymes, such as superoxide dismutase (SOD), catalase (CAT), and ascorbate peroxidase (APX), tend to perform better under abiotic stresses, such as water deficit [27,35,36,37,38]. However, this study evaluated total antioxidant activity through DPPH antioxidant assay, which does not account for the specific effect of each enzymatic or non-enzymatic antioxidant defense mechanism. 

DPPH is considered to be a model of a stable lipophilic radical. Antioxidants react with DPPH, reducing the number of DPPH free radicals to the number of their available hydroxyl groups [39]. The amount of DPPH free radical scavenged accounted for the total amount of antioxidants present in the sample. Using DPPH assay, antioxidant activity in extracts from the sugarcane leaves of each genotype varies, suggesting a difference in the total antioxidant activity and ROS-reducing capacity of each variety [39]. An increase in concentrations of several antioxidants (i.e., CAT and APX) during drought stress, especially during the tillering or early vegetative stages of sugarcane, have been observed [33]. These past findings are consistent with this study’s results, which observed higher scavenging activity during drought at the tillering stage. Furthermore, increases in antioxidant concentration (CAT, peroxidase, and glutathione reductase) during drought at different stages of the growth of maize were significantly different across genotypes. It was also observed that maize exposure to drought in its early stages of growth (4 to 5 leaf stage) leads to higher antioxidant activity compared to drought exposure during the anthesis and grain filling stages [34]. 

Membrane damage due to oxidative stress could be evaluated by measuring the electrolyte leakage of leaf samples. During drought or heat stress, membrane permeability increases; therefore, electrolytes and ions can easily diffuse [23]. Measuring the electrical conductivity could help assess the relative membrane injury of the leaf samples. Results showed an increase in the EC values of leaf samples from the drought-stressed plots. These results agree with previous studies in sugarcane [20,40], which found that elevated levels of electrolyte leakage were indicative of relative injury in sensitive sugarcane genotypes during moisture stress conditions. Results also suggest that most of the genotypes (i.e., PHIL 2006-1899, PHIL 2000-1419, and PHIL 2000-0791) with higher levels (between 62.6–64.5%) of AOA tend to have lower EC (between 49.1–56.33%) compared to genotypes PHIL 2004-1011, PHIL 2005-1763, and PHIL 2003-1389, with AOA values ranging from 52.7% to 55.6% and corresponding EC values ranging from 52.8% to 60.8%. This trend is also evident in the Pearson’s correlation analysis showing a significant negative correlation between AOA and EC. This relationship was also observed in durum wheat [41], coconut [42], and maize [34], providing further indication of the variability of EC leakage values between stress-tolerant and susceptible genotypes.

In general, higher EC values can be observed in the stalk elongation plots relative to the tillering plots. Interestingly, plants from the stalk elongation plots have significantly lower AOA concentration than the tillering plots. Differences in electrolyte leakage at different developmental stages of maize and sorghum have been reported, and results showed that pre-flowering dehydration produced higher electrolyte leakage values than post-flowering dehydration, which suggest an apparent difference in AOA activities during both of the stages of growth [43]. 

Another widely used indicator of drought response is stomatal conductance. Reduction in stomatal conductance is the immediate response of plants during water deficit conditions, and serves as an indicator of effective plant signaling during stressed conditions [44]. Significant stomatal conductance reduction during stress could affect photosynthetic processes, such as gas exchange [40]. Changes in photosynthetic rates by stomatal regulation not only limit gas exchange capacity but also affects CO_2_ assimilation that could result in a series of photochemical reactions that lead to oxidative damage to the photosynthetic systems of the plant [45]. Drought-tolerant genotypes have been observed to have mechanisms to adjust to these particular conditions, such as efficient osmotic adjustment, as well as better antioxidant systems [22]. This study showed that in general, drought reduced the stomatal conductance of all genotypes evaluated, however, significant variation among genotypes under stress was observed for both growth stages. Also, the maintenance of stomatal conductance close to the non-stress level is genotype- and growth stage-dependent. Sugarcane genotypes experienced a significant reduction under drought [8,46]. Correlation analysis also showed a significant negative correlation between stomatal conductance and AOA, which was consistent with other drought-related studies in sugarcane [45]. 

The drought tolerance indicators also correlated with different agronomic traits related to yield. It was evident in the results that plant height and internode length were both significantly affected by drought. However, the effect of drought among genotypes varied, which could be attributed to the different mechanisms previously discussed. These results were consistent with previous drought experiments [6,47,48]. Similar results focusing on the effect of drought on several cane attributes and its correlation with each other have also been reported [17].

On the other hand, this study established a significant correlation between plant height and plant physiological parameters for each growth stage of stress imposition, with r values varying for each formative stage. This was the same with the internode length and the physiological parameters; however, it was only significant during the tillering stage and not during the stalk elongation stage. Drought has a higher impact on dry matter accumulation and leaf area during the tillering stage, which could be supported by the observed effects of drought on the physiological characteristics of sugarcane during the same developmental stage [49]. Additionally, both stalk height and internode length ultimately dictate the yield of sugarcane; therefore, any decrease in these parameters will result in a reduction in the final yield [40].

Stalk diameter values for each genotype were also significantly different from the highest average values in PHIL 2155 (33.81 mm) and the lowest values in PHIL 2004-1011 (26.82 mm). Variation in stalk diameter under drought stress was mostly affected by genotype [48], which is evident in the present study as no significant differences between the formative stages were observed. Correlation between AOA and Gs was present during the stalk elongation while Chl and Gs during the tillering stage. However, both have relatively low coefficient values (0.26–0.30). Drought conditions affected stalk height to a greater extent than stalk diameter [47]. 

For the tiller count, the average values among genotypes were not significantly different. However, a significant reduction was observed in the average number of tillers in the drought stress treatment (5.24) in comparison with the control (5.92). The number of tillers was already observed to be significantly reduced in sugarcane plants subjected to drought stress during their formative stages [30]; this includes tillering and the grand growth stage (stalk elongation stage). Higher tiller count was also observed in the tillering stage (6.40) than the stalk elongation stage (4.76). Similarly, water deficit during the earlier developmental stage of sugarcane tends to promote tillering in order to compensate for the reduced assimilation production during drought [49].

PCA during each of the formative stages produced different sets of groupings, as well as varying contribution values of each parameter. At stalk elongation, the majority of the contributions in PC1 were from EC (17.26), Gs (15.33), plant height (20.51) and stalk diameter (25.51 while 83.58% of the total contributions to the PC2 came from Chl 34.05), AOA (29.06) and Gs, (20.47) while at the tillering stage, Chl (22.62), plant height (23.94), and EC (17.79) contribute highly to PC1, and for PC3, Chl (20.35), AOA (20.75), Gs (15.31), and stalk diameter (30.82) were the major contributors. Most of the physiological parameters were identified as a major contributor to at least one principal component. These parameters were most likely to have the most effect on the overall variation of the data set and could be identified as significant or key contributors to the drought response of each sugarcane genotype during stalk elongation. Higher loadings/contributions of each parameter suggest that the following has the strongest association within each PC, and thus could be used as a selection criterion for selection and breeding for drought tolerance [50]. Different studies also observed the efficiency of utilizing several physiological parameters in discriminating sugarcane genotypes, as well as different crops [51,52,53,54].

Furthermore, examining each cluster revealed the similarities in the agro-physiological response of each genotype belonging in the same group at each formative stage. One interesting trend observed in both formative stages was that significantly higher Gs during water deficit conditions appear to have better agronomic characteristics and lower EC values, as well as higher AOA. It is already known how significant stomatal conductance is during drought conditions [55]. Reduced photosynthetic rate, as well as stomatal conductance during drought, resulted in low levels of internal CO_2_ concentration due to inhibition of photosynthetic enzymes such as Rubisco [45,56]. This decrease in photosynthetic capacity could limit the growth and development of sugarcane genotypes during drought resulting in poor agronomic performance.

However, PCA-based bi-plots of each experiment showed different clustering for each of the sugarcane genotypes. A possible explanation was the differences in the PCA loading values for each of the developmental stages, as well as changes in the tolerance capability of a genotype as it grows and develops. Sugarcane genotypes at later stages, such as the stalk elongation stage, were more tolerant to drought compared to earlier developmental stages, such as the tillering stage [56]. 

This observation provides a possible explanation for the changes in the groupings for both of the formative stages.

## 4. Materials and Methods

### 4.1. Experimental Treatment and Planting Materials

Two field experiments were conducted at the Institute of Plant Breeding-Central Experimental Station, College of Agriculture and Food Science, University of the Philippines Los Baños (14°8′ N, 238°44′ W, 36 masl) to evaluate the response of 10 high-yielding sugarcane varieties to water deficit imposed at two different growth stages after ratooning. Two fields were planted at different planting dates but grown under similar environmental conditions to synchronize data gathering and treatment imposition. Drought imposition was done by withdrawing irrigation water from the water-deficit plots, coinciding with the target growth stage of the crop. These stages were the tillering stage at two months after ratooning and stalk elongation at five months after ratooning. 

The experimental design of each field trial followed a split-plot design in a randomized complete block design with three replicates per genotype. Two water regimes were assigned as whole plots, and sugarcane genotypes were assigned as sub-plots. Each genotype was planted in six 9 m long rows, 1 m apart, maintaining 36 plants per row. The 10 sugarcane genotypes used in the experiments were identified and recommended by the Sugar Regulatory Administration, Department of Agriculture, Philippines, as high-yielding varieties. 

### 4.2. Meteorological Conditions and Field Moisture Level

**Figure 4:**
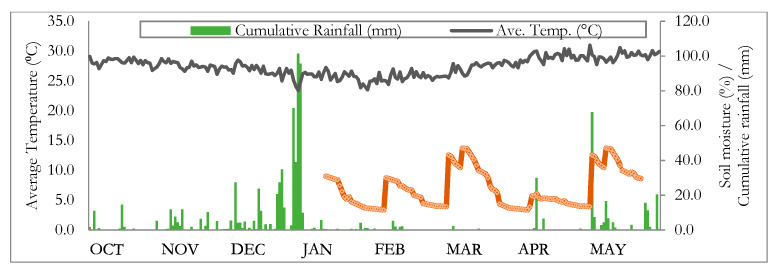
Monthly average temperature, cumulative rainfall, and soil moisture (red line) of the experimental field from October 2018 to April 2019. Drought stress treatment started in March 2019.

Different meteorological parameters were monitored throughout the experiment. Soil moisture was determined weekly at the start of drought imposition using an HH2 soil moisture meter (Model ML3, Delta-T Devices, Burwell, Cambridge, UK). Meter readings were done at a soil depth of 25–30 cm. Data for relative humidity and amount of rainfall were also gathered from the UPLB-National Agromet Station (14°19′ N, 238°44′ W, 28 masl) throughout the experiment. The average temperature in the field from October 2018 to May 2019 ranged from 23.4 °C to 31.0 °C, and the cumulative rainfall during the imposition of drought (March to April 2019) was about 41 mm (Figure 4). Soil moisture in the well-watered plots was maintained at field capacity (28–30%), while drought-stressed plots reached 11.59% during the peak of stress. 

### 4.3. Data Collection

Physiological parameters and agronomic traits were only collected from 10 selected plants located in the central rows for each genotype per replicate, for a total of 30 plants per genotype. Data were measured at the peak of drought stress at 30 days after treatment imposition for both experiments. All physiological parameters were measured from the second fully expanded leaf of each selected plant.

#### 4.3.1. Total Chlorophyll Content

The chlorophyll content of dried plant tissue was evaluated using a modified procedure [57]. The leaf samples collected were oven-dried for 48 h at 65 °C. Dried samples were powdered using an ED-5 Wiley mill. Powdered samples at 50 mg were used, and 4 mL of 80% aqueous acetone (v/v) was added to the powdered sample contained in 13 × 100 mm test tubes. The mixture was then shaken for 5 min and then centrifuged for another 5 min. The extracts were then transferred into a new test tube and adjusted to 15 mL by adding 80% aqueous acetone. The total chlorophyll content was obtained using the protocol by Mackinney [58]. Absorbance readings of the sample extracts were determined under 645 nm (OD645) and 663 nm (OD663) using a Shimadzu UV-Vis spectrometer (Model UV-1280). Total chlorophyll content was computed as follows:

*Total chlorophyll content (Chl) = (20.2 × OD645) + (8.02 × OD663)* (1)

#### 4.3.2. Total Scavenging Activity

Total antioxidant activity was determined using DPPH radical scavenging assay. DPPH radical is typically used for determining the free radical scavenging ability of natural compounds. Compounds or extracts were allowed to react with a stable free radical, DPPH (2,2-diphenyl-1-picrylhydrazyl), and the reduction of DPPH due to reaction with antioxidants will result in a decrease in absorbance at a specific characteristic wavelength of the solution [59]. Following the protocol designed by Chiappero et al. [60], with slight modification, 200 mg leaf samples were used to obtain 1 mL leaf extract. The leaf samples were added to 5 mL 80% methanol and were incubated for only 30 min (vortexed every 10 min) instead of 90 min. After the incubation, the samples were centrifuged, and the resulting supernatant was obtained. Then, 1.0 mL of the supernatant was added to 2.9 mL DPPH and was incubated in the dark for another 30 min at room temperature. Absorbance was measured at 517 nm with the DPPH solution as blank. Scavenging activity was computed using the formula:

(2) % scavenging activity={[1−(ODblank−OD517)/ODblank]}×100.

#### 4.3.3. Electrolyte Leakage

For the evaluation of electrolyte leakage (%), leaf disks were collected from the second fully expanded leaf of five plants from each plot. The leaf discs were washed with nano-pure water three times to remove dirt and other external sources of electrolytes, as mentioned in [61]. The samples were placed in test tubes with 10 mL distilled water and incubated for 22 h at room temperature on a rotary shaker at 100 rpm. The initial electrolyte (EC_initial_) reading was measured after incubation. Following a modified version of the procedure in [62], samples were placed in a hot water bath at 90 °C for 1 h. Another electrolyte reading (EC_final_) was taken after samples were cooled at room temperature. All readings were made using an Ionix pocket conductivity tester (Model EC20). Electrolyte leakage was computed as

(3) $$EC=\frac{EC_{initial}}{EC_{final}}.$$

#### 4.3.4. Stomatal Conductance

Stomatal conductance measurements were taken from the second fully expanded leaf from the top of each plant and measured weekly throughout the duration of drought condition using a Delta-T portable leaf porometer (Model AP4). 

#### 4.3.5. Agronomic Traits

Plant height was measured from the base of the plant to the last node at the top. Stalk diameter was measured at three different portions of the stem (top, middle, base) using a digital caliper. Nodes were manually counted to calculate the average internode length of each plant sample. The number of tillers per plant was also recorded. 

### 4.4. Statistical Analysis

The data were subjected to ANOVA using SAS Studio statistical software with the standard error set at 5% level of significance. Mean comparisons were evaluated based on Tukey’s range test (honestly significant differences; HSD). Correlation analyses using Pearson’s correlation coefficients were also done in order to describe the trait-by-trait relationship. Lastly, principal component analysis (PCA) was performed using princomp() function in ggplot2 [63] and factoextra [64], in order to determine the contribution of each parameter to the overall variation in the response of the sugarcane genotypes during a specific growth stage and to visualize the differences and similarities of the sample population using R version 3.6.2 [65]. PCA, which has been described as one of the most widely used multivariate analyses to assess differences and similarities in a data set [66], was used to describe the overall variation of a multivariate data set by transforming each original variable, in this case, the physiological and agronomic parameters, into a new set of specific linear combinations of principal components (PCs). It was used to classify and identify genotypes using PCs that obtained from the original parameters and were tested on different crops, such as cherry [67], wheat [68], sorghum [69], sesame [70], and rice [71,72] using morpho-physiological parameters. 

## 5. Conclusions

Results from this study indicate the observable response of sugarcane genotypes subjected to drought at different formative stages. The physiological parameters and agronomic traits investigated in this study could easily be measured and utilized as key selection criteria for drought tolerance breeding for sugarcane. PCA and correlation analysis of the data demonstrates that Chl, AOA, EC, and Gs all have a significant relationship with at least one of the agronomic traits, at either or both developmental stage. Biplots utilizing the highest contributing PCs for each developmental stage were also able to group genotypes with similar characteristics to verify the effectiveness of the parameters used. Changes in the groupings of the genotypes in the two developmental stages demonstrate the changes in the response of each genotype at each stage of growth, as well as the changes in the relationship of physiological and agronomic parameters. Information gathered from these experiments showed that some physiological parameters might be suitable only at a particular developmental stage and, therefore, should be taken into consideration when performing genotype selection for drought tolerance breeding in sugarcane. 

## Figures and Tables

**Table 1:** Analysis of variance, means, and Tukey’s honest significant differences (HSD) for total chlorophyll content (Chl), scavenging activity (AOA), electrolyte leakage (EC), stomatal conductance (Gs), plant height (PH), stalk diameter (SD), internode length (IL), and tiller count (TIL) grown under drought condition and well-watered condition at tillering and stalk elongation stages.

Treatment	Physiological Traits								Agronomic Characteristics							
	*Chl*		*AOA*		*EC*		*Gs*		*PH*		*IL*		*SD*		*TIL*	
Genotype																
PHIL 2006-2289	9.35	a	58.47	abcd	48.83	b	88.09	a	95.53	ab	11.47	ab	28.83	ab	5.96	a
PHIL 2006-1899	8.14	abc	64.51	a	56.33	ab	98.60	a	98.67	ab	10.87	abc	28.83	ab	4.93	a
PHIL 2005-1763	9.22	ab	52.68	cd	52.67	ab	111.38	a	115.52	ab	10.34	abc	28.56	ab	5.54	a
PHIL 2004-1011	6.66	c	55.61	bcd	60.75	a	89.18	a	118.16	a	11.53	ab	26.82	b	5.53	a
PHIL 2003-1389	7.71	abc	51.00	d	52.42	ab	110.67	a	104.07	ab	12.48	a	27.49	ab	5.96	a
PHIL 2000-2569	7.21	bc	58.00	abcd	59.33	a	101.90	a	94.56	ab	8.46	c	28.89	ab	6.13	a
PHIL 2000-1419	6.94	bc	62.61	ab	49.08	b	91.48	a	103.32	ab	11.88	ab	28.02	ab	5.92	a
PHIL 2000-0791	7.35	bc	64.25	a	52.58	ab	116.96	a	89.45	b	9.65	bc	28.01	ab	5.7	a
PHIL 8013	6.97	bc	62.37	ab	57.50	a	94.87	a	107.90	ab	10.52	abc	28.07	ab	5.14	a
PHIL 2155	7.55	abc	60.74	abc	55.75	ab	101.50	a	118.77	a	11.19	abc	33.81	a	5.01	a
Water Treatment																
Drought-stressed	5.58	b	67.44	a	61.78	a	92.42	b	80.51	b	97.74	b	30.72	a	5.24	b
Well-watered	9.84	a	50.61	b	47.27	b	108.5	a	124.54	a	111.94	a	26.74	b	5.92	a
Formative Stage																
Tillering (2 MAR)	6.76	b	69.97	a	49.55	b	99.47	a	76.66	b	8.82	b	28.10	a	6.40	a
Elongation (5 MAR)	8.66	a	48.08	b	59.50	a	101.45	a	128.40	a	12.86	a	29.37	a	4.76	b
*F*- Value																
Genotype (G)	3.29	**	6.76	**	4.99	**	2.30	*	3.26	**	3.74	**	1.60		2.40	*
Treatment (E)	172.04	**	213.17	**	158.46	**	14.97	**	157.50	**	33.26	**	17.52	**	15.24	**
Formative Stage (T)	34.52	**	361.26	**	74.44	**	0.23		210.54	**	112.80	**	1.79	**	86.47	**
G × E	0.89		2.03	*	2.64	**	4.11	**	1.07		1.37	**	1.27		0.49	
G × T	3.55	**	6.5	**	3.93	*	864.73	**	1.85		5.32	**	0.71		2.95	**
E × T	15.23	**	46.54	**	7.46	**	5.17	**	10.55	**	13.41		2.84		6.72	*
G × E × T	2.67	**	7.33	**	2.75	**	8.71	**	1.46		2.27	*	0.86		1.51	
CV (%)	19.07		10.69		11.58		22.67		18.99		19.26		18.10		17.23	

Means with the same letter within the same column per trait/characteristic, are not significantly different at 5% level of significance. MAR = months after ratooning. * *p* < 0.05; ** *p* < 0.01.

**Table 2:** Correlation analysis of the physiological traits and morphological characteristics of the different sugarcane genotypes during the tillering stage.

Source	Chl	AOA	EC	Gs	PH	IL	SD	TIL
Chl		0.452 **	−0.611 **	−0.631 **	0.607 **	0.594 **	0.378 **	0.159
AOA			−0.327 *	−0.567 **	−0.486 **	−0.449 **	−0.223	−0.068
EC				−0.572 **	−0.391 **	−0.414 **	−0.237	−0.198
Gs					−0.672 **	−0.582 **	−0.279 *	−0.166

Notes: Total chlorophyll content (Chl), scavenging activity (AOA), electrolyte leakage (EC), Scavenging activity (AOA), stomatal conductance (Gs), plant height (PH), stalk diameter (SD), internode length (IL), and tiller count (TIL); * *p* < 0.05; ** *p* < 0.01.

**Table 3:** Correlation analysis of the physiological traits and morphological characteristics of the different sugarcane genotypes during the stalk elongation stage.

Source	Chl	AOA	EC	Gs	PH	IL	SD	TIL
Chl		0.532 **	−0.473 **	−0.653 **	0.377 **	0.045	0.203	0.522 **
AOA			−0.366 **	−0.585 **	−0.297 *	−0.039	−0.382 **	−0.333 **
EC				−0.626 **	−0.432 **	−0.170	−0.235	−0.368 *
Gs					−0.511 **	−0.191	−0.261 *	−0.454 **

Notes: Total chlorophyll content (Chl), scavenging activity (AOA), electrolyte leakage (EC), stomatal conductance (Gs), plant height (PH), stalk diameter (SD), internode length (IL), and tiller count (TIL); * *p* < 0.05; ** *p* < 0.01.

**Table 4:** Principal component analysis showing eigenvectors, eigenvalues, and percent variance of physiological and agronomic traits of 10 sugarcane genotypes under drought at the tillering stage.

Parameters	PC1	PC2	PC3	PC4	PC5
Chl	−0.4756	0.2322	−0.4511	0.0602	−0.1448
AOA	0.3644	0.2738	0.4555	−0.4717	−0.2302
EC	0.4218	−0.4136	−0.3497	−0.0741	0.6024
Gs	−0.3684	−0.3487	0.3913	−0.4275	0.2798
Plant height	−0.4893	−0.3525	−0.0639	−0.3564	−0.0968
Stalk diameter	−0.2025	−0.1339	0.5552	0.6594	0.1998
Internode length	−0.2165	0.6605	0.0358	−0.1543	0.6600
Tiller count	−0.4756	0.2322	−0.4511	0.0602	−0.1448
Standard deviation	1.526	1.317	1.201	0.952	0.479
Proportion of variance, %	33.3	24.8	20.6	13.0	3.3
Cumulative proportion, %	33.3	58.1	78.6	91.6	94.8

**Table 5:** Principal component analysis showing eigenvectors, eigenvalues, and percent variance of physiological and agronomic traits of 10 sugarcane genotypes under drought at stalk elongation stage.

Parameters	PC1	PC2	PC3	PC4	PC5
Chl	−0.2067	−0.5835	0.3382	0.4163	−0.1025
AOA	0.2089	0.5390	0.5106	0.0708	−0.5971
EC	−0.4154	0.1083	−0.4335	−0.4953	−0.3152
Gs	0.3916	−0.4525	−0.0787	−0.2745	−0.5248
Plant height	0.4528	0.0325	0.3042	−0.4796	0.4923
Stalk diameter	0.5051	−0.2573	−0.2809	0.0085	−0.1185
Internode length	−0.3570	−0.2920	0.5093	−0.5205	−0.0421
Tiller count	−0.2067	−0.5835	0.3382	0.4163	−0.1025
Standard deviation	1.717	1.286	0.975	0.909	0.602
Proportion of variance, %	42.1	23.6	13.6	11.8	5.2
Cumulative proportion, %	42.1	65.7	79.3	91.1	96.3

## Appendix A. Supplementary Data

**Table A1:** Total chlorophyll content of the 10 sugarcane genotypes subjected to drought during tillering and stalk elongation stages.

Variety	Total Chlorophyll Content (mg/g)							
	Tillering Stage				Stalk Elongation Stage			
	Well-Watered		Drought		Well-Watered		Drought	
PHIL 2006-2289	12.04	ab	12.04	ab	12.04	ab	12.04	ab
PHIL 2006-1899	15.29	a	15.29	a	15.29	a	15.29	a
PHIL 2005-1763	14.19	ab	14.19	ab	14.19	ab	14.19	ab
PHIL 2004-1011	7.75	c	7.75	c	7.75	c	7.75	c
PHIL 2003-1389	12.32	bc	12.32	bc	12.32	bc	12.32	bc
PHIL 2000-2569	10.58	ab	10.58	ab	10.58	ab	10.58	ab
PHIL 2000-1419	7.64	c	7.64	c	7.64	c	7.64	c
PHIL 2000-0791	11.67	abc	11.67	abc	11.67	abc	11.67	abc
PHIL 8013	11.74	abc	11.74	abc	11.74	abc	11.74	abc
PHIL 2155	11.06	bc	11.06	bc	11.06	bc	11.06	bc

Means with the same letter, within the same column per trait/characteristic, are not significantly different at 5% level of significance.

**Table A2:** Scavenging activity of the 10 sugarcane genotypes subjected to drought during tillering and stalk elongation stages.

Variety	Total Scavenging Ability (%)							
	Tillering Stage				Stalk Elongation Stage			
	Well-Watered		Drought		Well-Watered		Drought	
**PHIL 2006-2289**	**54.17**	**def**	**90.40**	a	47.88	a	41.41	b
PHIL 2006-1899	74.15	ab	81.44	ab	45.20	a	59.56	a
PHIL 2005-1763	42.35	fg	73.32	b	44.54	a	50.32	ab
PHIL 2004-1011	46.36	efg	80.05	ab	42.88	a	52.71	ab
PHIL 2003-1389	31.08	g	76.79	ab	42.88	a	49.68	ab
PHIL 2000-2569	56.82	cdef	83.17	ab	43.25	a	50.76	ab
PHIL 2000-1419	58.57	bcde	91.29	a	44.35	a	55.46	ab
PHIL 2000-0791	75.99	ab	87.51	ab	43.84	a	51.52	ab
PHIL 8013	71.23	abc	71.93	b	42.90	a	62.04	a
PHIL 2155	65.61	abcd	87.26	ab	43.34	a	51.06	ab

Means with the same letter, within the same column per trait/characteristic, are not significantly different at 5% level of significance.

**Table A3:** Electrolyte leakage of the 10 sugarcane genotypes subjected to drought during tillering and stalk elongation stages.

Variety	Electrolyte Leakage (%)							
	Tillering Stage				Stalk Elongation Stage			
	Well-Watered		Drought		Well-Watered		Drought	
PHIL 2006-2289	42.92	a	47.11	b	59.05	ab	64.19	b
PHIL 2006-1899	52.09	a	71.62	ab	55.5	ab	67.52	b
PHIL 2005-1763	41.16	a	53.09	b	52.91	ab	65.24	b
PHIL 2004-1011	48.06	a	53.88	b	53.1	ab	85.43	a
PHIL 2003-1389	37.65	a	50.67	b	39.81	b	70.48	ab
PHIL 2000-2569	42.69	a	50.17	b	57.86	ab	62.86	b
PHIL 2000-1419	49.69	a	71.45	ab	47.7	ab	64.41	b
PHIL 2000-0791	47.33	a	57.91	ab	52.29	ab	68.10	b
PHIL 8013	36.68	a	46.37	b	40.25	b	74.67	ab
PHIL 2155	46.88	a	57.52	ab	55.33	ab	69.33	ab

Means with the same letter, within the same column per trait/characteristic, are not significantly different at 5% level of significance.

**Table A4:** Stomatal conductance of the 10 sugarcane genotypes subjected to drought during tillering and stalk elongation stages.

Variety	Stomatal Conductance (mmol/m/sec)							
	Tillering Stage				Stalk Elongation Stage			
	Well-Watered		Drought		Well-Watered		Drought	
PHIL 2006-2289	122.83	a	104.67	a	180.33	b	37.70	ab
PHIL 2006-1899	169.33	a	16.40	b	139.17	b	31.80	b
PHIL 2005-1763	149.83	a	41.83	b	190.83	b	60.17	ab
PHIL 2004-1011	154.5	a	51.33	ab	186.00	b	15.77	ab
PHIL 2003-1389	146.00	a	106.67	a	68.62	c	44.63	c
PHIL 2000-2569	158.17	a	43.17	b	240.33	a	26.18	ab
PHIL 2000-1419	149.67	a	44.17	b	170.60	b	15.03	b
PHIL 2000-0791	148.83	a	23.20	b	192.23	b	41.72	ab
PHIL 8013	160.67	a	16.13	b	154.00	b	21.55	b
PHIL 2155	165.83	a	16.23	b	184.17	b	28.15	ab

Means with the same letter, within the same column per trait/characteristic, are not significantly different at 5% level of significance.
